# One-Bed-One-Team—Does an Integrated General Hospital Inpatient Model Improve Care Outcomes and Productivity: An Observational Study

**DOI:** 10.3389/fpubh.2022.779910

**Published:** 2022-03-04

**Authors:** Yi Feng Lai, Shi Qi Lee, Yi-Roe Tan, Zheng Yi Lau, Jason Phua, See Meng Khoo, Satya Pavan Kumar Gollamudi, Cher Wee Lim, Yee Wei Lim

**Affiliations:** ^1^MOH Office for Healthcare Transformation, Singapore, Singapore; ^2^Department of Pharmacy, Alexandra Hospital, Singapore, Singapore; ^3^Department of Pharmacy, National University of Singapore, Singapore, Singapore; ^4^School of Public Health, University of Illinois at Chicago, Chicago, IL, United States; ^5^Division of Policy Research and Evaluation, Ministry of Health, Singapore, Singapore; ^6^Alexandra Hospital, National University Health System, Singapore, Singapore; ^7^Department of Medicine, Yong Loo Lin School of Medicine, National University of Singapore, Singapore, Singapore

**Keywords:** integrated care, acuity transition, inpatient, care coordination, generalist

## Abstract

**Introduction:**

With the increasing complexity of healthcare problems worldwide, the demand for better-coordinated care delivery is on the rise. However, current hospital-based practices remain largely disease-centric and specialist-driven, resulting in fragmented care. This study aimed to evaluate the effectiveness and feasibility of an integrated general hospital (IGH) inpatient care model.

**Methods:**

Retrospective analysis of medical records between June 2018 and August 2019 compared patients admitted under the IGH model and patients receiving usual care in public hospitals. The IGH model managed patients from one location with a multidisciplinary team, performing needs-based care transition utilizing acuity tagging to match the intensity of care to illness acuity.

**Results:**

5,000 episodes of IGH care entered analysis. In the absence of care transition in intervention and control, IGH average length of stay (ALOS) was 0.7 days shorter than control. In the group with care transition in intervention but not in control, IGH acute ALOS was 2 days shorter, whereas subacute ALOS was 4.8 days longer. In the presence of care transition in intervention and control, IGH acute ALOS was 6.4 and 10.2 days shorter and subacute ALOS was 15.8 and 26.9 days shorter compared with patients under usual care at acute hospitals with and without co-located community hospitals, respectively. The 30- and 60-days readmission rates of IGH patients were marginally higher than usual care, though not clinically significant.

**Discussions:**

The IGH care model maybe associated with shorter ALOS of inpatients and optimize resource allocation and service utilization. Patients with dynamic acuity transition benefited from a seamless care transition process.

## Introduction

Globally, the rapidly aging population with a growing chronic disease prevalence has placed an increasing burden on healthcare systems (1, 2). Healthcare systems are facing the challenge of caring for patients with multimorbidity. The prevalence of multimorbidity increases with age, affecting over 60% of people aged 65 years or older (3–6). With the increasing complexity of multimorbidity, the demand for a comprehensive and coordinated delivery of healthcare services is expected to rise, and healthcare expenditure is expected to soar with inpatient load as the main cost driver (7–9). However, current hospital-based practices remain largely disease-centric with a specialist focus. This is partly driven by the population's preference to seek care from specialists with the belief that they provide better enhanced quality of care (10, 11). Consequently, individuals have multiple specialists managing different health problems during their inpatient stay, resulting in fragmented care and an inefficient hospital system.

To tackle this changing healthcare landscape, integrated care models have been implemented in many countries (12). Integrated care is generally defined as care which involves greater coordination of health and social services, leading to seamless and holistic management (13). Beyond process reorganization, integrated care places the needs of an individual, a family and a community at its center, shifting the approach from disease-centric to person-centric. For the context of this paper, we examined the inpatient aspect of integrated care, in which acute and subacute care are integrated; hence, patients are managed at one setting by one multidisciplinary team, usually led by a generalist (14, 15). Integrated care programmes have been shown to improve the quality of care, patient and caregiver satisfaction, access and cost (12, 16).

In Singapore, the government has implemented various initiatives over the years to provide highly efficient and enhanced coordinated care. These initiatives include reorganization of healthcare services into three integrated regional health systems, increasing a primary care system's capacity and establishing national registries (17, 18). The regional health systems were established to help reduce care fragmentation by including institutions which span the entire health continuum, from acute hospitals (AH) to community hospitals (CH), in each cluster. The government envisioned a seamless transition across different care settings via shared clinical pathways (19, 20). Nevertheless, having acute and subacute care in separate institutions means that patients still need to be transferred between different settings when they transit between episodic acute care and subacute rehabilitative care. Care across multiple sites not only leads to transfer delays and avoidable bed-days, but gaps in knowledge transfer and information sharing also exist between the sites and care teams (17).

The current ecosystem in Singapore still focuses largely on episodic care (21). To meet the evolving complex needs of a population with a finite pool of manpower resources and infrastructure capacities, new care models which offer consolidated care in a highly efficient manner should be explored. Although evidence indicates varying extents of success of integrated care models, most studies were based in the United States and Europe, with limited studies in Asia (12). The current study evaluated a programme in which patients received integrated care in a public hospital in Singapore. Findings from this study could prove useful for other hospitals attempting to implement such a care model in a similar context.

## Methods

### Settings

#### “FAST” Programme

Since June 2018, “FAST” wards in Alexandra Hospital, National University Health System, have operated on the basis of the integrated general hospital (IGH) model which encourages patients to move away from seeking care from multiple specialists. The one-bed-one-team approach is adopted, and patients are managed from one location by a multidisciplinary team across the entire care continuum. This approach is made possible by integrating acute and subacute inpatient care within the same ward. A generalist-led team assesses patient acuity at regular intervals, and the amount of clinical resources devoted is adjusted accordingly in a dynamic manner. The acuity level of a patient is loosely defined as L3 for acute care, L2 for subacute care and L1 for rehab care, with L3 being equivalent to AH care and L2/L1 being equivalent to CH care under the usual care model. Lower acuity levels receive less contact time with physicians (L1<L2<L3) but still receive daily management by a nursing team with a lower intensity of monitoring. The intention is to ensure that resources are allocated in an efficient and cost-effective manner and patient care is uncompromised.

#### Usual Care

By contrast, usual care separates acute and subacute care, with some hospitals having CHs co-located within the same premise as an AH, whereas most hospitals do not. For AHs that do not have co-located CHs, transfers of care between the institutions are usually more cumbersome. In addition, patients with multimorbidity are usually managed by separate specialists instead of one multidisciplinary team. This has inadvertently resulted in the fragmentation of care, in which patients must be transferred from one team to another or from one facility to another as they progress through their illness. Whilst IGH optimizes resources by having nurse-led teams manage patients with lower acuity, nurses in usual care play a more passive role in patient management.

### Study Design

Electronic medical records of patients, including demographic variables, clinical indicators and financial factors, were extracted from Ministry of Health's national casemix datasets. Further detailed financing and costs data were extracted from National University Health System, one of the three regional health systems in Singapore, which included data from IGH wards in Alexandra Hospital, usual care general medicine wards in National University Hospital (NUH, without co-located CH) and usual care general medicine wards in Ng Teng Fong General Hospital (NTFGH, with co-located Jurong Community Hospital, JCH). All data were de-identified prior to analysis.

Comparisons were planned between IGH patients and two separate groups of controls- Usual care with co-located CHs and usual care without co-located CHs.

For phase one of analysis for primary outcomes, Ministry of Health's national casemix datasets from June 2018 to March 2019 were used. Patients admitted under the IGH wards were compared with patients receiving usual care with data included in the de-identified national cohort from the datasets. Propensity score matching was performed using variables that included age, gender, race, residence type, primary diagnosis, Charlson Comorbidity Index and procedures undergone during hospitalization.

For phase two of analysis for secondary outcomes, financing and costs data from National University Health System between July 2018 and August 2019 were used. Patients admitted under the IGH wards in Alexandra Hospital were compared with patient cohorts from general medicine wards in NUH and NTFGH. Propensity score matching was also performed using variables that included age, gender, race, residence type, primary diagnosis, Charlson Comorbidity Index and procedures undergone during hospitalization.

Prior to propensity score matching, there were a total of 7,087 cases and more than 500,000 controls from national casemix databases where both control groups were selected from. After propensity score matching, a total of 5,000 episodes of IGH care, 9,078 controls without co-located CHs and 7,919 controls with co-located CHs entered analysis. In our propensity score matching, unmatched IGH patients and controls were discarded from the analysis. The quality of matching was evaluated by analyzing the standardized percentage bias across the covariates, which was satisfactorily and significantly reduced post-matching.

### Data Analysis

Patient demographics and baseline characteristics were summarized through descriptive statistics. Primary outcome measures included the average length of stay (ALOS) and 30- and 60-days readmission rates, which were compared between the matched intervention group and two groups of usual care controls: (1) patients who received care in hospitals with co-located CH and (2) patients who received care in hospitals without co-located CH. Differences in ALOS between intervention and control were calculated in number of days and compared through *T*-test for each of the following categories: (1) absence of care transition (patients with only L3 acuity level in intervention vs.patients with only AH stay in control); (2) heterogeneous care transition (patients with L3 to L2/L1 acuity transition in intervention vs. patients eligible for acuity transition but not transferred to CH in control) and (3) presence of care transition (patients with L3 to L2/L1 acuity transition in intervention vs. patients transferred to CH in control). Differences in 30- and 60-days readmission rates were compared using one-way ANOVA.

Secondary outcome measures included productivity and cost measures, specifically discharge rates to step-down care services/acuity transition rates, hospitalization costs and service utilization. These were performed among a subset of selected cases and controls were further matched based on their care trajectories- (1) patients without acuity transition, (2) patients with acuity transition in IGH but no CH transfer in controls, and (3) patients with acuity transition in IGH and CH transfer in controls. Differences in care transition rates were reported in proportions, hospitalization costs were analyzed to identify cost drivers in intervention and control, and inpatient service utilization was reported in ratios. Financial data showed that the main cost drivers of hospitalization were room charges, investigations and daily treatment fee. We used the hospitals' daily treatment fees instead of hospitalization bill sizes as the surrogate measure to account for manpower and equipment resourcing because other fixed costs including room charges accounted for in gross hospitalization bills were insignificantly different across institutions. The differences in investigation costs in the current analysis were also attributable to economies of scale and logistics irrelevant for care model comparisons. All statistical analyses were performed with Stata version 16 with *P* < 0.05 regarded as significant.

### Ethics Approval

This study was approved by the National Healthcare Group Domain Specific Review Board (Ref: 2020/00023).

## Results

### Patient Characteristics

The characteristics of patients under IGH care and the two subgroups of usual care controls are presented in Table 1.

**Table 1:** Demographics of patients in different care models.

**Patient characteristics**	**Usual care (without CH co-location)**	**Usual care (with CH co-location)**	**IGH patients**
No. of patients	9,078	7,919	5,000
Age, mean	69.0	68.1	70.4
Gender = Male	4,176 (46%)	3,722 (47%)	2,350 (47%)
**Ethnicity** Chinese Malay Indian Others	6,264 (69%) 999 (11%) 999 (11%) 817 (9%)	5,306 (67%) 1,029 (13%) 871 (11%) 713 (9%)	3,400 (68%) 600 (12%) 550 (11%) 450 (9%)
**Housing** 1–2 rooms flat 3–4 rooms flat 5-rooms and executive flat Private housing	1,362 (15%) 5,356 (59%) 1,543 (17%) 817 (9%)	1,109 (14%) 4,672 (59%) 1,584 (20%) 554 (7%)	800 (16%) 2,950 (59%) 850 (17%) 400 (8%)
Average CCI score	3.2	3.2	3.2
**Among those with SGO data** Live alone Little/No interaction with family (less than once a month) Mobility-impaired Need assistance to doctor	545 (6%) 272 (3%) 1,634 (18%) 2,633 (29%)	317 (4%) 158 (2%) 1,267 (16%) 2,138 (27%)	350 (7%) 200 (4%) 800 (16%) 1,450 (29%)

*IGH, Integrated General Hospital; CH, Community Hospital; CCI, Charlson Comorbidity Index; SGO, Silver Generation Office*.

### Primary Outcomes

As shown in Table 2, though the 30- and 60-days AH readmission rates of IGH patients were marginally higher than those of patients under usual care, the values were not statistically or clinically significant.

**Table 2:** Readmission rates comparison between intervention and usual care.

**Clinical outcomes**	**Variables**	**Usual care (without CH co-location)**	**Usual care (with CH co-location)**
AH readmission rates within 30 days	IGH	16.5%	16.2%
	Comparator	15.9%	14.6%
	Difference	+0.6%	+1.6%
AH readmission rates within 60 days	IGH	22.8%	22.6%
	Comparator	23.2%	20.6%
	Difference	−0.4%	+2.0%

*IGH, Integrated General Hospital; AH, Acute Hospital; CH, Community Hospital*.

**Figure 1:**
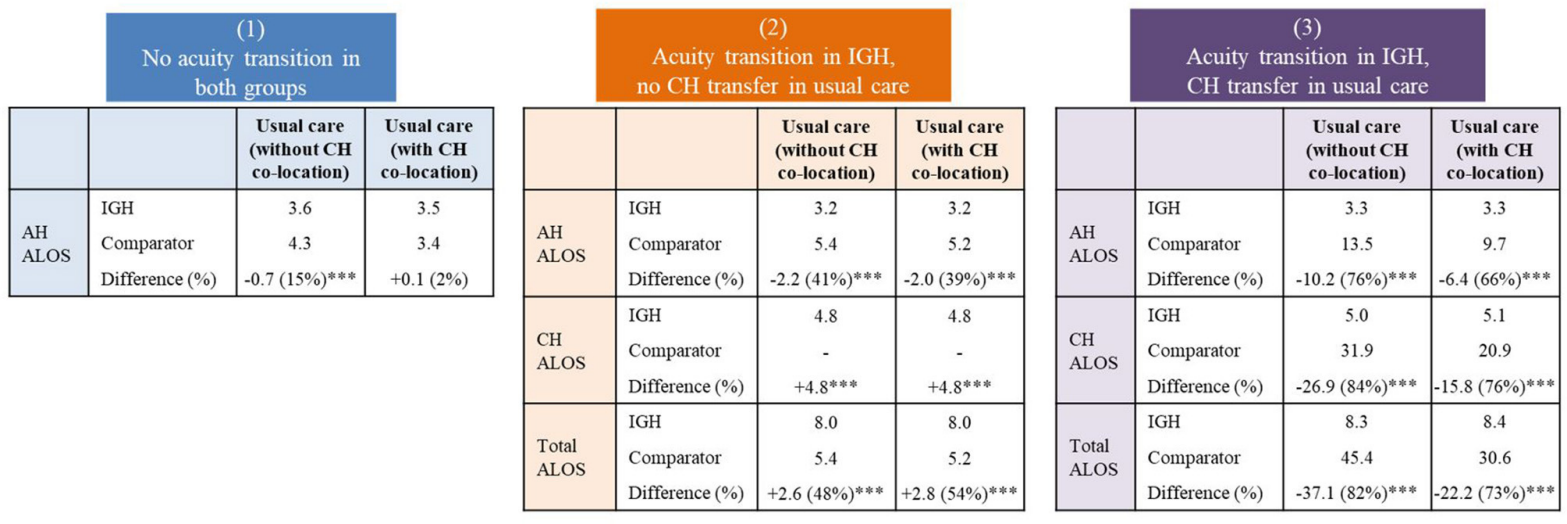
Average length of stay (days) comparison between intervention and usual care. IGH, Integrated General Hospital; AH, Acute Hospital; CH, Community Hospital; ALOS, Average Length of Stay. ^***^P < 0.001.

Figure 1 shows the ALOS comparison between IGH patients and controls. Among patients without acuity transition, the IGH acute care episodes (AH ALOS) was 0.7 days shorter than that of patients under usual care in hospitals without CH co-location. Among patients with acuity transition in IGH but no CH transfer in controls, the IGH AH ALOS was 2 days shorter, but subacute care episodes (CH ALOS) was 4.8 days longer, indicating that for every AH day saved, there was a corresponding additional two lower resourced CH days expended (with an ALOS trade-off ratio of 1:2). Among patients with acuity transition in IGH and CH transfers in controls, the IGH AH ALOS was 10.2 and 6.4 days shorter and the CH ALOS was 26.9 and 15.8 days shorter compared with those of patients under usual care in hospitals without CH co-location and with CH co-location, respectively.

### Secondary Outcomes

The majority of IGH patients had medical diagnoses that were commensurate with a significant proportion of regular general medicine patients at other AHs. However, significant differences in acuity transition rates existed. For the same diagnosis code, 41% of the patients in IGH underwent acuity transition from L3 to L2/L1, whereas only 1% of the patients under usual care were discharged from AHs to CHs.

Table 3 shows the cost and productivity analyses categorized by acuity transition. Cost categories included medication, investigation, treatment services, consumables and consultation fees accounting for approximately 30% of total costs. Amongst all episodes, IGH and NUH's costs were comparable (1%), whereas NTFGH's cost was significantly lower (22%). Among patients without acuity transition, the IGH cost per episode was 8% lower than that for usual care at NTFGH and 34% lower than that at NUH. This difference was ascribed to the lower IGH ALOS (by 3 and 13%, respectively) and lower unit cost for IGH service items, especially for daily treatment fee.

**Table 3:** Cost and productivity analyses between intervention and usual care.

	**IGH (Intervention)**	**NUH (Usual care without co-located CH**	**NTFGH (Usual care with co-located CH)**
**Overall cost comparisons**			
No. of matched episodes	1,182		
Cost per episode (% difference)	$5,249	$5,315 (+1%)	$4,075 (−22%)
**Among patients without acuity transition**			
No. of matched episodes	619		
Cost per episode (% difference)	$3,247	$4,340 (+34%)	$3,515 (+8%)
**Among patients with acuity transition in IGH but no CH transfer in controls**			
No. of matched episodes	595		
Cost per episode (% difference)	$7,307	$5,370 (−27%)	$4,509 (−38%)
**Among patients with acuity transition in IGH and CH transfer in controls**			
No. of matched episodes	68 (vs. NUH) 112 (vs. NTFGH)	68	112
Cost per episode (% difference)	$9,907 (vs. NUH) $12,891 (vs. NTFGH)	$25,958 (+162%)	$19,785 (+53%)
**Productivity comparisons**			
	**IGH**	**NUH**	**NTFGH**
**Daily treatment fee**			
L1	$111	$280	$256
L2	$135		
L3	$169		
***ALOS***			
Acute hospital/L3	3.2	5.4	5.2
Community hospital/L1.L2	4.8	-	-
**Overall cost**			
L3 cost	$540.80 (3.2days x $169)	$1,512 (5.4days x $280)	$1,331.20 (5.2days x $256)
L1/L2 cost	$590.40 (4.8days x $123)	-	-
Total cost (% difference)	$1,131.20	$1,512 (+33.7%)	$1,331.20 (+17.7%)

*IGH, Integrated General Hospital; NUH, National University Hospital; NTFGH, Ng Teng Fong General Hospital; CH, Community Hospital; ALOS, Average Length of Stay*.

Among patients with acuity transition in IGH but no CH transfer in controls, the IGH overall cost per episode was 38% higher than that for usual care at NTFGH and 27% higher than that at NUH. When analyzed by variable costs and care model productivity by using daily treatment fee as surrogate, the IGH cost was then 17.7 and 33.7% lower than those at NTFGH and NUH, respectively, primarily because of the lower L3 daily cost (Table 3). The cost trade-off ratio was ~1:2, with one L3 day under usual care equivalent to two L1/L2 days under the IGH model. This cost trade-off ratio was similar to the ALOS trade-off ratio of 1:2 indicated above. Thus, limited savings were achieved from a lower IGH daily treatment fee. The relatively modest productivity gain was also negated by larger cost drivers, such as room charges, resulting in an overall higher hospitalization cost for IGH. Among patients with acuity transition in IGH and CH transfer in controls, the IGH overall cost per episode was 53% lower than that for usual care at NTFGH and 162% lower than that at NUH, primarily driven by the much higher CH ALOS for NUH and NTFGH patients.

## Discussion

### Multi-Element Integrated Care Model

Many integrated care models have been implemented and evaluated internationally (22). Studies which examined the length of stay as an outcome have shown inconsistent evidence, with some reporting a reduction, whereas others found an insignificant effect (12). In our study, we found that the IGH inpatient model resulted in an overall shorter ALOS. This finding could be due to several differentiating elements of the IGH model.

Firstly, having one care team to overlook a patient's entire care continuum in the hospital instead of having multiple specialists was proven more efficient without the need for case handovers and duplicative investigations. Secondly, the IGH model reduced administrative hassle and transfer delays by managing patients from one location without the need to move patients physically across institutions, resulting in timely and frictionless care transition. Lastly, a seamless acuity transition process allowed regular adjustment of patient acuity when a patient's condition improved or deteriorated during the care episode, contributing to a smooth continuity of care. The interplay of these elements contributed to decreased ALOS in IGH patients and reduced cost per episode. This finding has been similarly demonstrated in other studies that incorporated multiple elements into their integrated care models (23).

### Resource Optimisation Through Smooth Acuity Transition

Our study found that for the same diagnosis code, much lesser patients in usual care underwent care transition compared with those under IGH care. This group of patients reflects the potential pool of patients that an IGH model could have an effect on because the only current mechanism to influence acuity transition in usual care systematically is through an AH–CH transfer. However, this mechanism is seldom meaningfully practiced for vast majority of patients due to barriers in transfer, complex transfer application and documentation and operational and financial frameworks at CHs (24, 25). Hence, the implementation of an IGH model could result in process optimisation and resource savings by targeting patients in a high-risk group who have a high likelihood of undergoing acuity transition. As demonstrated in the IGH model and in the literature, this group could include patients who have multimorbidity, low socioeconomic status and mobility impairment and are older and living alone (26).

For patients requiring acuity transition, the current ALOS trade-off ratio is similar to the cost trade-off ratio at 1:2, negating the effects of any productivity gains and causing the overall hospitalization cost to be high for IGH. The ALOS and cost trade-off ratios should be maximized to make IGH financially favorable compared with usual care. This objective can be achieved through workforce substitution and increased efficiency in care transition. A multidisciplinary team can efficiently match patients' care plans to their illness trajectories by familiarizing with the acuity tagging system. This strategy allows service utilization to be tagged to the right acuity levels and clinical resources to be allocated in an efficient manner (14). Communication within the care team and administrative protocols can also be improved to achieve enhanced efficiency in care transition across acuity levels (27). To yield further cost savings, an IGH model may be explored in CH-like settings and resources instead of acute care settings.

Lessons from this study suggested that the areas where holistic integrated care models such as IGH model could provide the greatest benefits are in whole-of-person care. Such an approach reduced fragmentation and improved discharge planning especially for the increasing proportion of general medicine patients not requiring specialist care as a result of aging population and rising chronic diseases. These patients could be looked after by a generalist care team to allow for cost-effective single-team holistic management.

### Strengths and Limitations

The strengths of this study are that the data were extracted from the national cohort and a comprehensive analysis was conducted on a nationwide level including all public hospitals. This study is also one of the few integrated care programmes in the region which looked at the use of acuity labeling and its effects on care outcomes and productivity. The limitations are that patients' disease severity was not an outright variable in the propensity score matching, considering that no standardized measurement is available for disease severity across institutions. Some of the large differences in ALOS observed, especially in group 3 comparison, could be magnified due to this poor matching of disease severity. We tried to account for this limitation by including variables which are equivalent predictors, such as the Charlson Comorbidity Index, primary diagnosis and procedures performed during the length of stay. In addition, while other analyses such as cost-effectiveness could be useful to be carried out, this retrospective study was limited by the amount and type of extractable data available for review, with the aim of providing early signals to the care model validation. The team would be pursuing a follow-up prospective cohort study to further validate the care model outcomes during hospitalization, as well as potential benefits in post-discharge healthcare utilization.

### Implications and Outlook

Findings from this study contribute to the global pool of evidence validating this new model of care, especially in the Asian context where evidence is scarce. Given that we build insights into clinical efficiency, manpower sustainability and healthcare financing, policy owners can make a highly informed decision on hospital care redesign and whether such an integrated care model is feasible and sustainable to be developed as mainstream care in our ecosystem. Moving forward, the perspectives and acceptability of patients, caregivers and various stakeholders of this IGH model implementation should be explored. Beyond inpatient care and hospital-based measures, the short- and medium-term effects of the IGH care model could not be evaluated in this retrospective analysis. Future studies, such as a prospective cohort study, could provide additional insights into the downstream effects of IGH and the degree of care integration beyond hospital walls. Such areas as discharge planning, post-discharge care experience, care continuity and post-discharge utilization of healthcare resources are potential factors that will contribute to a comprehensive IGH model.

## Conclusion

The IGH care model has shown promising results in shortening the ALOS of inpatients by providing a holistic and coordinated management of their illnesses through a one-bed-one-team approach. The IGH model has the potential to allow for highly efficient care transition and resource optimisation without compromising on the quality of patient care by accurately and dynamically tagging a patient's trajectory of illness to appropriate acuity levels. Thus, general medicine patients who require acuity transition, which is the majority of inpatient load, should be the patient segment that the IGH model focuses on to become a mainstream care model that tackles the evolving complex healthcare needs of patients.

## Data Availability Statement

The datasets analyzed during the current study are available from the corresponding author on reasonable request.

## Ethics Statement

The studies involving human participants were reviewed and approved by National Healthcare Group Domain Specific Review Board. Written informed consent for participation was not required for this study in accordance with the national legislation and the institutional requirements.

## Author Contributions

YL, SL, JP, SK, SG, CL, and YWL were involved in the study conception and study design. JP and YL were involved in the extraction of data. SL and ZL were involved in data analysis. Y-RT, YL, JP, SK, SG, CL, and YL were involved in the interpretation of findings. Y-RT was involved in the writing of the manuscript. All authors participated in the review of the manuscript, provided critical revisions, and gave the final approval of the version to be published.

## Funding

This research was internally funded by the Ministry of Health Office for Healthcare Transformation (MOHT) to support the research for Integrated General Hospital Care Model Evaluation in the National University Health System in Singapore.

## Conflict of Interest

The authors declare that the research was conducted in the absence of any commercial or financial relationships that could be construed as a potential conflict of interest.

## Publisher's Note

All claims expressed in this article are solely those of the authors and do not necessarily represent those of their affiliated organizations, or those of the publisher, the editors and the reviewers. Any product that may be evaluated in this article, or claim that may be made by its manufacturer, is not guaranteed or endorsed by the publisher.
